# A Database of Chinese-English Bilingual Speakers: Ratings of the Age of Acquisition and Familiarity

**DOI:** 10.3389/fpsyg.2020.554785

**Published:** 2020-09-18

**Authors:** Jue Wang, Baoguo Chen

**Affiliations:** Beijing Key Laboratory of Applied Experimental Psychology, Faculty of Psychology, Beijing Normal University, Beijing, China

**Keywords:** age of acquisition, second language, Chinese translation equivalents, familiarity, concreteness

## Abstract

Recently, considerable attention has been given to the effect of the age of acquisition (AoA) on learning a second language (L2); however, the scarcity of L2 AoA ratings has limited advancements in this field. We presented the ratings of L2 AoA in late, unbalanced Chinese-English bilingual speakers and collected the familiarity of the L2 and the corresponding Chinese translations of English words. In addition, to promote the cross-language comparison and motivate the AoA research on Chinese two-character words, data on AoA, familiarity, and concreteness of the first language (L1) were also collected from Chinese native speakers. We first reported the reliability of each rated variable. Then, we described the validity by the following three steps: the distributions of each rated variable were described, the correlations between these variables were calculated, and regression analyses were run. The results showed that AoA, familiarity, and concreteness were all significant predictors of lexical decision times. The word database can be used by researchers who are interested in AoA, familiarity, and concreteness in both the L1 and L2 of late, unbalanced Chinese-English bilingual speakers. The full database is freely available for research purposes.

## Introduction

The age of acquisition (AoA) refers to the age at which a word is acquired and is considered to be the fifth most important factor affecting lexical decision times, following word frequency, word length, similarity to other words, and word onset (Kuperman et al., 2012). Words learned early in life are processed faster than words learned late in life. Although this AoA effect has been studied for more than 60 years and has been explored in different populations and experimental tasks, its origin in language processing is still under debate. There are two major theories accounting for the mechanism of the AoA effect: the Semantic Hypothesis and the Arbitrary Mapping Hypothesis.

According to the Semantic Hypothesis, AoA reflects an intrinsic property of words’ semantic representations: words acquired early contain richer semantic connections and occupy a central position in the semantic network; therefore, they are easier to access than words acquired later (van Loon-Vervoorn, 1989; Brysbaert et al., 2000). If the first language (L1) and second language (L2) words share the same semantics, the L2 words should inherit the AoA characteristics of the corresponding L1 words; that is, the AoA effect in L2 reflects the order of the word meaning acquisition in L1 (Izura and Ellis, 2002). Only the L1 AoA effect can be observed, and no independent L2 AoA effect exists.

The Arbitrary Mapping Hypothesis is related to how connectionist networks learn and proposes that the information that enters the network earlier has a greater influence on the final network structure (Ellis and Lambon Ralph, 2000; Zevin and Seidenberg, 2002). The AoA effect reflects the arbitrary connections between input (e.g., orthography) and output representations (e.g., phonology and semantics). Therefore, different AoA effects are shown in different languages even when L1 and L2 words share the same semantic representations.

Clearly, the investigation of L2 AoA provides a new perspective for examining the mechanism of the AoA effect; however, studies of L2 AoA are very rare (Izura and Ellis, 2002, 2004; Hirsh et al., 2003; Dirix and Duyck, 2017; Xue et al., 2017). The paucity of L2 AoA research may be related to the lack of L2 AoA ratings. The current databases of AoA ratings are primarily in L1 (Gilhooly and Logie, 1980; Stadthagen-Gonzalez and Davis, 2006; Cortese and Khanna, 2008; Kuperman et al., 2012; Brysbaert et al., 2014), except for the study by Dirix and Duyck (2017), who collected L2 AoA ratings of unbalanced Dutch-English bilingual speakers. To the best of our knowledge, there is currently no large L2 AoA database of Chinese-English bilingual speakers; therefore, in Study 1, we collected L2 AoA ratings of unbalanced Chinese-English bilingual speakers by asking a limited number of participants to rate 1916 words (Moors et al., 2013; Brysbaert et al., 2014). We created the L2 AoA database for two reasons. First, the existence of an L2 AoA database may directly motivate the inspection of L2 AoA in word processing. This L2 AoA database not only provides a chance to study the L2 AoA itself but also facilitates the investigation of the relationship between L2 AoA and other variables, such as L1 AoA and word frequency. Importantly, the investigation of L2 AoA is helpful to clarify the origin of the AoA effect. Second, by enabling the exploration of whether AoA effects are influenced by the age at which an individual starts learning a language, the inclusion of L2 AoA ratings is useful for the verification of the critical period hypothesis of language acquisition (Hirsh et al., 2003).

In addition, we rated familiarity, which is considered in Study 1 to be the subjective frequency by which the English word frequency of Chinese-English bilingual speakers is measured. The Chinese translations of English words were also collected in Study 1. After determining the Chinese translation equivalents, in Study 2, we continued collecting the AoA ratings of the Chinese translation equivalents (two-character words). We collected these AoA ratings of Chinese translation equivalents for two reasons. First, we hoped to facilitate the cross-language research of AoA and not only the within-language research. As we mentioned before, the two theories explaining the AoA effects are controversial, especially when the semantics of L1 and L2 words are identical. The collection of the AoA ratings of the L1 translation words makes it possible to study whether the L2 AoA effect could exist independently of L1 AoA, the collection of which is crucial for understanding the mechanism of AoA. Second, two-character words are the most common word type in Chinese, and approximately 72% of Chinese words are of this type (Lexicon of Common Words in Contemporary Chinese Research Team, 2008). Thus, the collection of the AoA ratings of two-character words may be helpful for exploring the AoA effect in learning Chinese two-character words. The previous studies on the AoA effect on Chinese words all focus on single-character words (Chen et al., 2007, 2009; Liu et al., 2007, 2011; You et al., 2009). In addition to the collection of the AoA ratings of Chinese two-character words, due to the confusion caused by the higher correlation of AoA with familiarity and concreteness (Xue et al., 2017), in Study 2, we also collected data about familiarity and concreteness.

In summary, in Study 1, the present study includes the ratings of L2 AoA, L2 familiarity, and the corresponding Chinese translations of English words. After determining the Chinese equivalents, in Study 2, we continued to collect the ratings of L1 AoA, L1 familiarity, and the L1 concreteness of Chinese two-character words.

## Study 1

In Study 1, the ratings of L2 AoA and L2 familiarity, together with the Chinese translations of English words by unbalanced Chinese-English bilingual speakers, were gathered and are available in the Supplementary Material.

### Methods

#### Participants

The participants comprised 24 Chinese (L1)-English (L2) bilingual speakers, who completed L2 AoA ratings, and an additional 24 L1-L2 bilingual speakers, who participated in the rating of L2 familiarity and in the writing of the corresponding Chinese translation. The data from two participants were eliminated before the L2 AoA validation (in one case because the participant was a balanced bilingual speaker and in one case because of the lower correlation of the participant’s L2 AoA with the average L2 AoA value, i.e., a participant L2 AoA correlation beyond the average correlation plus or minus three standard deviations). The data from four participants were eliminated before the L2 familiarity validation because of the lower correlation with the average L2 familiarity value (i.e., a participant correlation beyond the average correlation plus or minus three standard deviations). All 42 participants were born in China, had no immigration or overseas education experience in their background, and had a mean age of 20.14 years (*SD* = 1.37, range: 18–24 years). The participants were paid and were recruited from several universities in Beijing. English is part of the school curriculum for all children in China. English courses are offered from grade 1 to grade 3; therefore, as children, the participants had started to learn English mainly from the ages of 7 to 9 years and had been studying English for a mean duration of 11.07 years (*SD* = 2.13, range: 7–15 years). The participants were unaware of the research purpose, were right-handed, and had normal or corrected-to-normal vision. The participants signed a consent form before the study. This study was approved by the Ethics Committee of the Faculty of Psychology, Beijing Normal University.

To access the English and Chinese proficiency of these 42 participants, self-assessment ratings were used to measure English and Chinese listening, speaking, reading, and writing skills on a scale that ranged from 1 (very poor) to 6 (excellent). Across the four aspects, the indicator by which English and Chinese proficiency was measured was the average score, and higher scores indicated a higher proficiency. The English proficiency of the participants was moderate (*M* = 3.45, *SD* = 0.75, range: 2.25–5.00) and lower than their Chinese proficiency (*M* = 5.20, *SD* = 0.55, range: 4.00–6.00). Additionally, English proficiency was also assessed by the Oxford Placement Test (OPT; Allan, 2004), which is considered a standard test to measure English proficiency. The OPT comprises 25 multiple-choice questions and a cloze test; the total achievable OPT score is 50. The mean OPT score was 39.36 (*SD* = 3.82, range: 31–46), and the split-half reliability coefficient was 0.76. Generally, the participants were late, unbalanced Chinese-English bilingual speakers.

#### Materials

For use as the experimental materials, a total of 1916 English words were selected according to the following criteria. First, the words were taken from English textbooks used in the third-year primary school to third-year senior school classes in China. The English textbooks were published by the People’s Education Press, which is affiliated with the Ministry of Education in China. Second, word frequency as indexed by SUBTLEX-UK (van Heuven et al., 2014) was considered, and a frequency range of 0.10 to 3611.87 per million (*M* = 61.57, *SD* = 186.53) was used. Third, we mainly focused on nouns; therefore, a noun was the part of speech that all the words could be used as. The ambiguous words that could be used in more than one grammatical category were words that might also be used as a verb and/or an adjective. Finally, we manually excluded words that could be potentially unfamiliar to participants.

For the L2 AoA ratings, the participants in this study were asked to rate the specific age (in years) at which they had learned the word, which was similar to other L2 AoA rating studies (Dirix and Duyck, 2017). We did not ask participants to use a 1–7 scale (Gilhooly and Gilhooly, 1979) because the onset and offset of L2 learning varies more than that of L1 learning and the L2 learning begins later and ends later; therefore, an age range including 1 as the minimum age and 13 as the older age cannot accurately capture the participants’ L2 word AoA. To improve the validity of the ratings, the stimulus lists beginning with 10 calibrator words along with the entire range of AoA were used to encourage participants to use the full range of values. Ten detection cells were also adopted (for example, please fill in 1 in this cell) in each of the lists, ensuring that the participants did not write random numbers. To ensure that the participants understood the instructions, we provided the following examples that included words that did not appear in the list: “If you think you learned ‘arm’ when you were 7 years old, please fill in 7. If you think you learned ‘jet’ when you were 12 years old, please fill in 12.”

For L2 familiarity ratings, the participants were required to rate their level of familiarity with the words by using a 1–7 scale, where higher numbers indicated a higher familiarity (Juhasz, 2008). The examples provided for this variable were as follows: “If you think that ‘arm’ is the most familiar, please choose 7. If you think that ‘jet’ is a little unfamiliar, please choose 3.” Moreover, these participants were also asked to write as many translations of the English words as possible. The translation of a word was determined only when the number of times the translation was provided greater than 70% of the total number of translations for the word.

#### Procedure

These 1916 words were divided into six lists of approximately 320 words each, and the order of these words was counterbalanced to avoid the sequence effect. Each participant was given an Excel file including four parts: study instructions, collection instructions, personal information section, and materials (all six lists, one for each sheet). The main instructions were reiterated in the header of each list. The participants were instructed to respond as accurately as possible but not to think too long. The participants could type in the letter N if they did not know the word well-enough to give an AoA rating. The participants were free to decide when to complete the ratings within 2 weeks, and they were told in advance that they would be paid only if their ratings were useful.

### Results

The values of L2 AoA were valid only when at least 70% of the raters gave numeric ratings rather than rating the words as unknown (similar to Izura and Ellis, 2002). In the data analysis, 81 words (4% of all words) were removed because of a lower recognition rate (less than 70%), which led to 1835 words that had reliable L2 AoA values (the average recognition rate was 98%). Each word’s AoA was rated by at least 16 participants (average: 22 participants; range: 16–22). To assess the L2 familiarity of these 1835 words, each word was rated by 20 participants, and the percentage of observations per word was 100%. The means, standard deviations, and ranges of the L2 AoA and the L2 familiarity of the 1835 words are presented in Table 1. The correlations between the two rated variables and the age of learning (AoL), word length, frequency, and concreteness are presented in Table 2. AoL refers to the time at which the learner is exposed to a word and denotes the time when a word first appears in standard school English textbooks (People’s Education Press, 2006; Xue et al., 2017). Following the results of the rating reliability, the characteristics of each rated variable were reported separately. Regression analyses were conducted to examine how each of these variables predicts the lexical decision times.

**TABLE 1:** Descriptive statistics for the ratings in Study 1.

**Rated variable**	**Minimum**	**Maximum**	**Mean**	***SD***
L2 AoA	9.55	17.06	13.59	1.30
L2 familiarity	3.05	7.00	6.02	0.73

*SD is the standard deviation. L2 AoA is the age of acquisition of a second language.*

**TABLE 2:** Correlations between the two rated variables and L2 AoL, length, frequency, and concreteness in Study 1.

	**L2 AoA**	**L2 AoL**	**L2 length**	**L2 frequency**	**L2 familiarity**	**L2 concreteness**
L2 AoA	1.00	0.76***	0.36***	−0.47***	−0.75***	−0.28***
L2 AoL		1.00	0.30***	−0.39***	−0.54***	−0.28***
L2 length			1.00	−0.38***	−0.11***	−0.36***
L2 frequency				1.00	0.51***	−0.06*
L2 familiarity					1.00	−0.09***
L2 concreteness						1.00

L2 AoA is the age of acquisition of a second language. L2 AoL is the time when a word first appears in standard school English textbooks. The frequency (log) was taken from the SUBTLEX-UK (van Heuven et al., 2014). Familiarity was rated on a 1–7 scale. The concreteness of the data was taken from Brysbaert et al. (2014). *p < 0.05; ***p < 0.001.

#### Reliability

To examine the reliability of the participants’ ratings, we calculated the split-half reliability coefficient (the correlation between the two groups to which participants were randomly assigned and that were as balanced as possible in terms of numbers) and two-way random intraclass correlation coefficient (ICC) for each of the rated variables. The ICC is the ratio of the true variance to the total variance, and the rest is random error. The value ranges from 0 to 1, where 0 indicates totally unreliable and 1 indicates perfectly reliable. For L2 AoA, the spilt-half reliability coefficient was 0.95, which was calculated by randomly dividing 22 participants into two groups. The ICC was 0.93, which was also high. For L2 familiarity, the spilt-half reliability coefficient was 0.95 and was assessed by randomly dividing 20 participants into two groups. The ICC was 0.94. These results indicated that among the raters, the reliability of L2 AoA and the familiarity ratings were high.

#### Rated Variable Characteristics

##### L2 AoA

**FIGURE 1:**
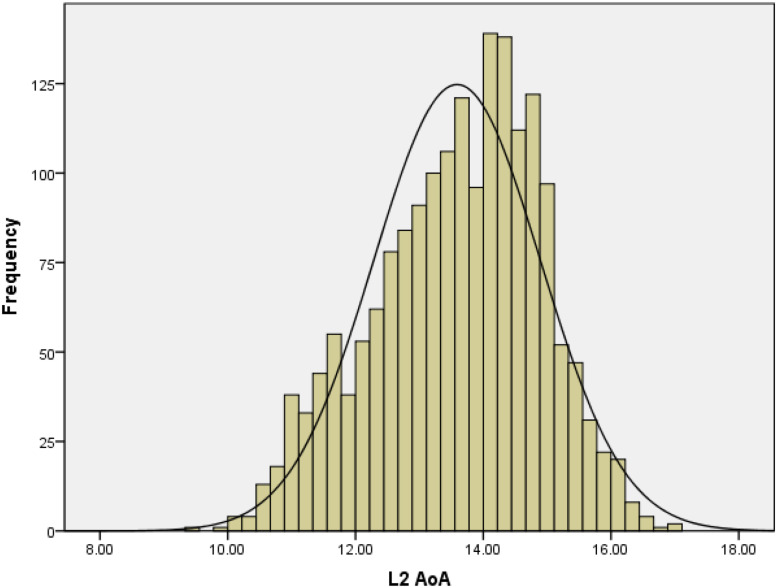
Frequency distribution of L2 AoA ratings in Study 1.

Figure 1 shows that the distribution of the L2 AoA ratings resembled a normal distribution (skewness is −0.34) similar to that in Dirix and Duyck (2017).

Table 2 reveals that L2 AoA is positively correlated with L2 AoL, word frequency, and familiarity and is negatively correlated with word length and concreteness. These correlations suggested that early acquired English words tended to be learned earlier in textbooks, were used more frequently, and were more familiar. In addition, these words were shorter and evoked concrete experiences.

To further validate the L2 AoA ratings, we correlated L2 AoA with the lexical decision times and then conducted simultaneous multiple regression analyses to explore the predictive power. First, we correlated L2 AoA with the lexical decision times of Berger et al. (2019; also seen in Skalicky et al., 2019). In that study, the lexical decision times for English words were collected by participants whose first language was not English. There were 1304 words in our data set that overlapped with those in Berger et al. (2019). The correlation between L2 AoA and the lexical decision times was 0.32, indicating that early acquired words were responded to faster. Then, we included L2 AoA, word length, and concreteness (from Brysbaert et al., 2014) as predictors in the regression models, and the largest variance inflation factor (VIF) was 1.21. The results of the regression model are displayed in Table 3 (adjusted *R*^2^ = 0.11). The L2 AoA can account for an extra 9% of the variance in lexical decision times after word length and concreteness are controlled for. However, it should be noted that as the participants’ proficiency level in Berger et al. (2019) was much higher than that in the present database, the predictive power might be smaller when L2 AoA correlations are made with the lexical decision times made by participants whose English proficiency level is also medium (Izura and Ellis, 2004). Next, we continued to validate L2 AoA through the analyses of lexical decision times that were made by medium-level Chinese English learners, and these lexical decision data are unpublished data collected by us. Among the 1835 English words for which L2 AoA values were collected in the present study, only 1154 words had lexical decision times.

**TABLE 3:** Results of regression analyses of L2 AoA on the lexical decision times.

**Variable**	Berger et al. (2019)			**Our data**		
	**β**	***SE***	***t***	**β**	***SE***	***t***
L2 AoA	0.32	1.57	11.36***	0.37	2.16	15.56***
L2 length	0.04	1.02	1.48	0.48	1.30	19.22***
L2 concreteness	0.02	2.00	0.65	0.19	2.79	7.99***

The variable codes are the same as in Table 2. ***p < 0.001.

The results of the regression model are again presented in Table 3 (adjusted *R*^2^ = 0.42), and the largest VIF was 1.22. The L2 AoA accounts for an extra 12% of the variance after word length and concreteness are controlled for. These results showed that the response times are shorter if the word is acquired earlier and that L2 AoA is the influential predictor of lexical decision times.

##### L2 familiarity

**FIGURE 2:**
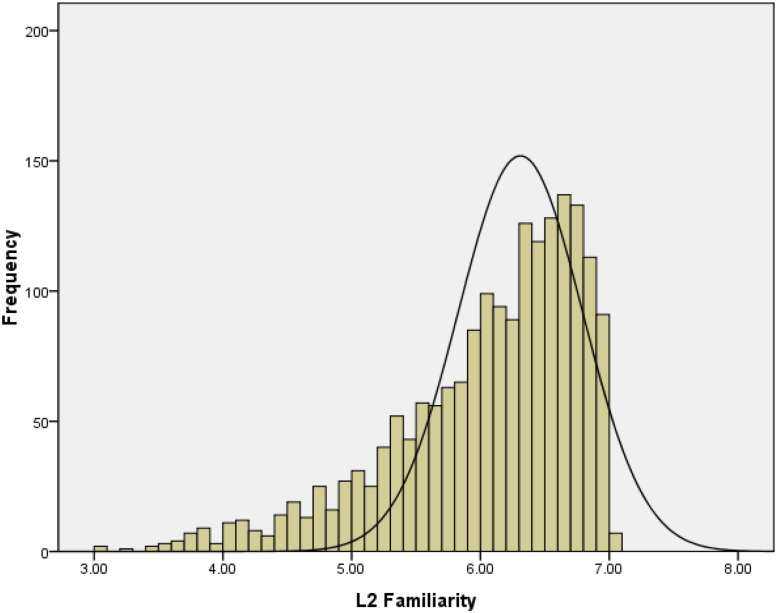
Frequency distribution of L2 familiarity ratings of Study 1.

Approximately 98% (1804/1835) of these words were rated above a 4.00 on a 1–7 scale, indicating that the participants were familiar with the majority of these English words. The distribution of the L2 familiarity ratings showed an obvious negative skewness of −1.12 (Figure 2).

Although statistically significant, the correlation between L2 familiarity and word frequency (the log of frequency from the SUBTLEX-UK; van Heuven et al., 2014) was moderate (see Table 2). The correlations between L2 familiarity and additional measures of word frequency (the log of frequency from the SUBTLEX-US; Brysbaert and New, 2009) were also calculated and found to be moderate (*r* = 0.54). The L2 familiarity was negatively correlated with several other variables, including age of learning (AoL), word length, and concreteness (see Table 2), which indicated that more familiar words tended to be learned early in the textbook, to be shorter, and to evoke concrete experiences.

Furthermore, we correlated L2 familiarity with the lexical decision times that we collected (unpublished data), and we ran regression analyses. We obtained the lexical decision times for only 1154 English words with L2 familiarity values. The correlation between L2 familiarity and the lexical decision times was −0.49. The results of the regression model are presented in Table 4 (adjusted *R*^2^ = 0.47), and the largest VIF was 1.17. L2 familiarity can account for an additional 16.5% of the variance in the lexical decision times after word length and concreteness are controlled for.

**TABLE 4:** Results of regression analyses of L2 familiarity on the lexical decision times.

**Variable**	**β**	***SE***	***t***
L2 length	0.51	1.22	21.71***
L2 concreteness	0.08	2.67	3.41***
L2 familiarity	–0.41	4.77	−18.84***

The variable codes are the same as in Table 2. ***p < 0.001.

##### Chinese translation

The Chinese translation was determined only when the number of times it was given as a translation was greater than 70% of the total number of translations; thus, 1317 English words had a matched Chinese meaning. The following criteria were used to select which of these 1317 Chinese translation equivalents were rated in Study 2: (a) only two-character translations were selected because the two-character word is the most common word type in Chinese and would therefore enable a better exploration of the Chinese AoA effect; (b) the word frequency of translations can be found in SUBTLEX-CH-WF (Cai and Brysbaert, 2010). This resulted in a remainder of 1053 Chinese two-character words, and the part of speech represented by these words was not necessarily a noun. In Study 2, we then collected the ratings of AoA, familiarity, and concreteness for these words.

In summary, in Study 1, we found the following: the distribution of L2 AoA and L2 familiarity ratings are similar to that for other AoA and familiarity ratings (Dirix and Duyck, 2017; Liu et al., 2018); the relation with other lexical variables meets expectations; and both variables are significant predictors of lexical decision times. For L2 words, the correlation between AoA and familiarity was significant and larger than the correlation between AoA and word frequency, confirming that the existing word frequency corpus created based on native English speakers is not fully appropriate to measure L2 words; therefore, familiarity should be taken into account, especially in L2 studies. Therefore, the ratings of L2 AoA and L2 familiarity are valid for measuring the age at which participants learned L2 words and to measure their familiarity with the L2 words.

## Study 2

In Study 2, we collected the ratings of AoA, familiarity, and concreteness for Chinese two-character words that are the translations of the English words from Study 1.

### Methods

#### Participants

A total of 60 native Chinese speakers completed the ratings of AoA, familiarity, and concreteness for L1 words, and each variable was rated by 20 participants. Because of the lower correlation with the average value (beyond the average correlation plus or minus three standard deviations), 2, 2, and 3 participants were eliminated prior to the data analysis of AoA, familiarity, and concreteness, respectively; thus, the AoA, familiarity, and concreteness analyses were conducted on a final set of 18, 18, 17 participants (*M* = 22.79 years, *SD* = 2.91, range: 18–28 years), respectively. The participants were from several universities in Beijing, had no background that included immigration or an overseas education experience, were right-handed, and had normal or corrected-to-normal vision. The participants were paid to participate, and all were unaware of the purpose of the study. Prior to the study, the participants signed a consent form. This study was approved by the Ethics Committee of the Faculty of Psychology, Beijing Normal University.

#### Materials

There were 1053 Chinese two-character words selected from Study 1. For L1 AoA ratings, the list preparation was largely the same as that used for the L2 AoA ratings, except for the 39 words of You et al. (2009); these 39 words were not included, and the remaining 1014 words were distributed across 4 lists. The order of these words were counterbalanced to avoid the sequence effect. To estimate L1 AoA, we used a 1–7 scale, where 2-year age bands were associated with a scale rating. For example, a rating of 1 meant the word was acquired at 1–2 years of age, a rating of 2 meant it was acquired at 3–4 years of age, and a rating of 7 meant the word was acquired at 13 years of age or older (Gilhooly and Gilhooly, 1979). Ten calibrator words were presented first in each list to improve the validity of the ratings, and 10 detection cells were included to avoid having the participants fill in random numbers. The examples given for this variable were as follows: “If you think you learned 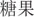 (candy) when you were 3 years old, please fill in 2. If you think you learned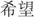 (hope) when you were 9 years old, please fill in 5.”

The familiarity instructions were the same as those in Study 1. A total of 466 words from Wang et al. (2019) were excluded, and the remaining 587 words were divided into 2 lists. The order of these words was counterbalanced. The concreteness ratings were based on a seven-point scale. Words that involved objects, materials, or people were more concrete, while words that involved abstract concepts were less concrete (Gilhooly and Logie, 1980). The 1053 words were divided into 4 lists, and the order was also counterbalanced. The examples provided for this dimension were as follows: “If you think 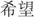 (hope) is the most abstract, please fill in 1; If you think 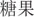 (candy) is the most concrete, please fill in 7.”

#### Procedure

The procedure was the same as that in Study 1. Each participant was given an Excel file that had a format similar to that of the Excel file used with the English words’ rating. The participants were given 10 days to finish the task.

### Results

There were 1014, 587, and 1053 words for which AoA, familiarity, and concreteness values, respectively, were collected. For the AoA, familiarity, and concreteness variables, the number of observations per word were 18, 18, and 17, and the percentage of observations per word was 100%. The means, standard deviations, and ranges of these variables are presented in Table 5. The correlations between the three rated variables and the number of strokes and frequency are presented for all 1053 words in Table 6. Strokes are the smallest constituent units of Chinese characters, and the number of strokes can be regarded as an indicator of the visual complexity of Chinese characters (Xing et al., 2004). Because early acquired words tend to have fewer numbers of strokes (Liu et al., 2007; Wang et al., 2019), we included the number of strokes in the correlation analysis. The rating reliability and the characteristics of each rated variable were described. Then, we presented the results of the regression analyses.

**TABLE 5:** Descriptive statistics for the ratings in Study 2.

**Rated variable**	**Minimum**	**Maximum**	**Mean**	***SD***
L1 AoA	1.33	6.89	4.46	0.98
L1 familiarity	3.65	6.60	5.43	0.56
L1 concreteness	2.18	6.88	4.74	1.26

*SD is the standard deviation. L1 AoA is the age of acquisition of the first language.*

**TABLE 6:** Correlations between the three rated variables and the number of strokes and frequency of all 1053 words in Study 2.

	**L1 AoA**	**L1 strokes**	**L1 frequency**	**L1 familiarity**	**L1 concreteness**
L1 AoA	1.00	0.13**	−0.18**	−0.48**	−0.45**
L1 strokes		1.00	−0.12*	–0.01	0.01
L1 frequency			1.00	0.28**	−0.14**
L1 familiarity				1.00	0.01
L1 concreteness					1.00

L1 AoA is the age of acquisition of the first language. Out of all the 1053 two-character words, 39 words’ L1 AoA values were taken from You et al. (2009), and 466 words’ familiarity values were taken from Wang et al. (2019). Table 6 presented the correlation results for all 1053 words, which were different from the specific correlation values of L1 AoA (1014 words) and L1 familiarity (587 words) in the text. The frequency (log) was taken from the SUBTLEX-CH-WF (Cai and Brysbaert, 2010). *p < 0.05; **p < 0.01.

#### Reliability

We assessed the reliability of the participants’ ratings by calculating the split-half reliability coefficient and the two-way random ICC for each of the rated variables. For the AoA ratings, by randomly dividing the 18 participants into two groups, the split-half reliability coefficient was found to be 0.96, and the ICC was 0.96. For the familiarity ratings, the split-half reliability coefficient and the ICC were 0.89 and 0.86, respectively; for the concreteness ratings, the split-half reliability coefficient and the ICC were 0.97 and 0.96, respectively.

#### Rated Variable Characteristics

##### L1 AoA

**FIGURE 3:**
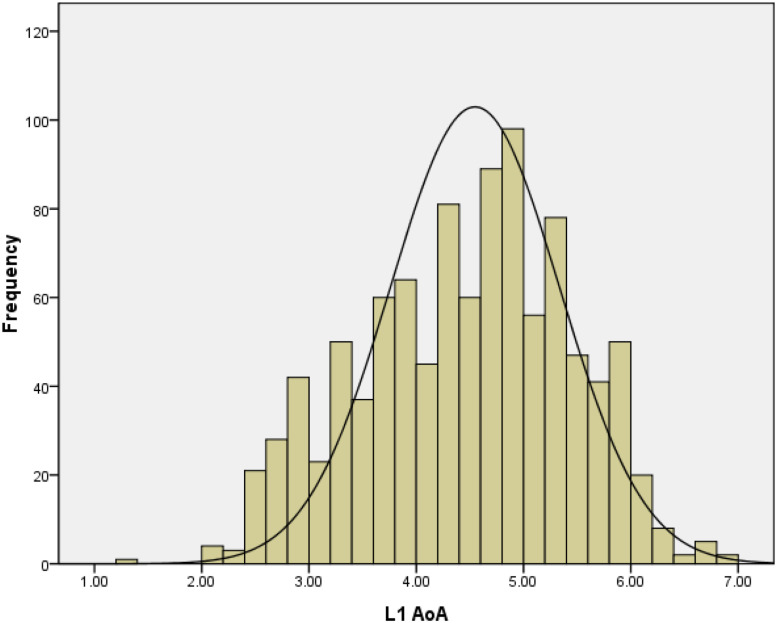
Frequency distribution of L1 AoA ratings in Study 2.

Figure 3 shows that the distribution of the 1014 words’ AoA ratings resembled a normal distribution (skewness is −0.21).

We examined the relation between L1 AoA and their corresponding English translation AoA (L2 AoA) and found that L1 AoA was moderately correlated with L2 AoA (*r* = 0.59); this correlation value is similar to that of Dirix and Duyck (2017, *r* = 0.52) and Ghyselinck et al. (2000, *r* = 0.60). The moderate correlations showed that the order of word acquisition in different languages is roughly the same but that differences also exist.

Although statistically significant, the correlation between AoA and word frequency (*r* = −0.18; the log of frequency from the SUBTLEX-CH-WF, Cai and Brysbaert, 2010) was low. To validate this result, we recalculated the correlation between AoA and the word frequency in Liu et al. (2011), who collected AoA ratings for the dominant names of 435 object pictures. It should be noticed that various types of words were included in Liu’s study. To make a better comparison with the present study, only two-character words were selected to reanalyze the correlation. We found the correlation was −0.21, which is a value similar to our correlation value. AoA is significantly correlated with familiarity (*r* = −0.48), concreteness (*r* = −0.44), and the number of strokes (*r* = 0.12). These results showed that early acquired L1 words tended to be more frequent and more familiar, evoked concrete experiences, and had fewer strokes.

To further validate our collected L1 AoA data, we compared our L1 AoA ratings with that of Liu et al. (2011). Between our dataset and that of Liu et al. (2011), there were 115 two-character words that overlapped, and the correlation between the two AoA ratings was 0.87.

##### L1 familiarity

**FIGURE 4:**
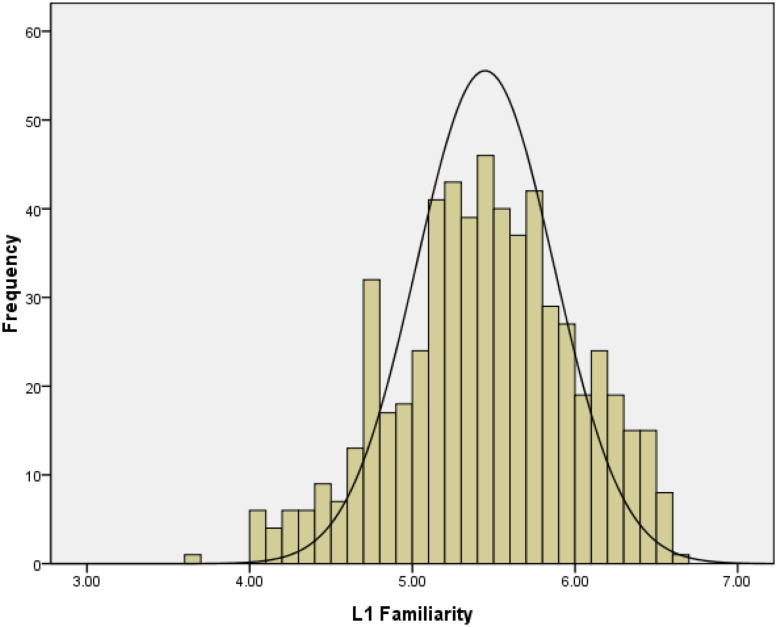
Frequency distribution of L1 familiarity ratings in Study 2.

Figure 4 presented the distribution of the 587 words’ familiarity ratings, which indicated that the participants were familiar with these words. We calculated the correlations between L1 familiarity and word frequency (*r* = 0.26; the log of frequency from the SUBTLEX-CH-WF, Cai and Brysbaert, 2010), and the correlation was found to be similar to that in Juhasz et al. (2015, *r* = 0.16). Moreover, L1 familiarity was significantly correlated with concreteness (*r* = 0.11) and the number of strokes (*r* = −0.16). More familiar words tended to be those that were rated as being more frequent, as evoking concrete experiences, and as having fewer strokes.

We also found a moderate correlation (*r* = 0.46) of the familiarity between L1 words and their English translations (L2 words), which indicated that the familiarity of the same concept expressed in Chinese and English is roughly similar but not completely the same.

##### L1 concreteness

**FIGURE 5:**
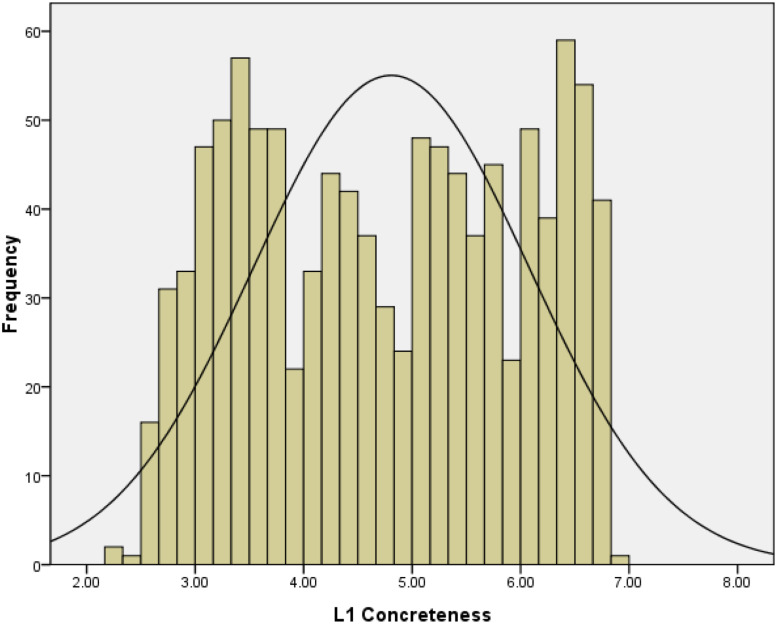
Frequency distribution of L1 concreteness ratings in Study 2.

Figure 5 shows the distribution of the 1053 words’ concreteness ratings. As previously mentioned, we found that more concrete words tended to be learned early in life and were more familiar. The specific correlation values are presented in Table 6.

Finally, we separately correlated the lexical decision times with the three rating variables (the 1014 words’ AoA, the 587 words’ familiarity, and the 1053 words’ concreteness) and then ran simultaneous multiple regression analyses. These lexical decision data are collected by us (unpublished data). The correlations were 0.24, −0.43, and 0.12, respectively. The regression results are shown in Table 7. We included AoA, familiarity, concreteness, number of strokes, and word frequency in the regression models (the largest VIF was 2.21). For the investigation of the L1 AoA’s predictive power, the adjusted *R*^2^ was 0.30. After controlling for the remaining four variables, the additional variance that can be accounted for by L1 AoA was 1%. For L1 familiarity and L1 concreteness, the adjusted *R*^2^ was 0.34 and 0.33, respectively. After controlling for the remaining four variables, L1 familiarity and L1 concreteness can explain an additional 6% and 2%, respectively, of the variance in the lexical decision times. The smaller influence of AoA was similar to that in Kuperman et al. (2012), who found that after controlling for inflected word forms, word frequency, word length, and the similarity to other words, 2.5% of the variance can be explained by AoA. Although the influence of these three variables was smaller, they still significantly predicted the lexical decision times.

**TABLE 7:** Results of the regression analyses of L1 AoA, L1 familiarity, and L1 concreteness on the lexical decision times.

**Variable**	**L1 AoA**			**L1 familiarity**			**L1 concreteness**		
	**β**	***SE***	***t***	**β**	***SE***	***t***	**β**	***SE***	***t***
L1 AoA	0.14	2.75	3.96***	0.09	3.72	1.87^+^	0.19	2.42	5.92***
L1 familiarity	–0.28	3.72	−8.86***	–0.33	6.04	−7.36***	–0.31	3.06	−11.05***
L1 concreteness	0.18	1.86	5.80***	0.17	2.48	4.23***	0.17	1.81	5.69***
L1 frequency	–0.04	0.02	–1.26	–0.02	0.05	–0.49	–0.03	0.02	–1.1
L1 strokes	0.36	0.43	13.35***	0.36	0.54	10.34***	0.34	0.41	13.09***

The variable codes are the same as those in Table 6. ^+^*p* < 0.1; ***p < 0.001.

In summary, the distribution of the AoA ratings resembled a normal distribution. The correlations between these variables are consistent with expectations, and all three ratings are significant predictors of the lexical decision times. Therefore, these ratings are considered valid measures.

## General Discussion

In the present study, we collected L2 AoA, L2 familiarity, and the corresponding Chinese translations of English words by unbalanced Chinese-English bilingual speakers. In addition, the L1 AoA, L1 familiarity, and L1 concreteness of Chinese two-character words were also obtained. We described the characteristics of each rated variable and evaluated the relationship between them. The correlation results showed that for English words, L2 AoA was positively correlated with AoL and word length and was negatively correlated with word frequency, familiarity, and concreteness. For Chinese two-character words, negative correlations of L1 AoA with word frequency, familiarity, and concreteness were found. Importantly, L1 AoA was moderately positively correlated with L2 AoA, indicating that word learning order roughly corresponds across languages but that two AoAs still can be distinguished, which provides an opportunity to further explore the relationship between them. The direction of these correlations is consistent with expectations. Words that are acquired later tend to be less familiar, less frequent, abstract, and longer.

We also found that familiarity is significantly correlated with AoA, concreteness, frequency, length, or number of strokes. Moreover, as previously noted, we also found that the correlation between AoA and the familiarity of L2 words is larger than the correlation between AoA and word frequency, suggesting these familiarity ratings may provide a better measure of the relative frequency of exposure to an L2 word than do objective measures of the printed word frequency of native speakers. For concreteness, concrete words are more familiar and tend to be acquired earlier. These results agree with the conclusions drawn in previous investigations (Juhasz and Rayner, 2003; Della Rosa et al., 2010) and offer support for the validity of the collected ratings.

The following describes the possible use of the present database. Generally, the present database includes a large number of words and rated variables, the availability of which encourages researchers to conduct mega studies, given the constraints of the limited materials used in orthogonal factor design; the present database may also make it convenient for researchers to select items that meet their requirements and routinely control irrelevant variables.

More specifically, the AoA data of L1 and L2 words that were translation word pairs were simultaneously collected for the first time. First, it is useful to further investigate AoA *per se*, for example, to examine whether AoA effects truly exist or are actually word frequency effects or cumulative-frequency effects in disguise. It is also important to investigate how the AoA effect differs in high and low proficiency levels (Perani et al., 1998). Second, the cross-language AoA database may encourage researchers to perform cross-language research, which makes it possible to compare the AoA effect across different languages. For instance, does AoA have the same influence on native speakers and L2 learners? Third, this database also helps to verify the Arbitrary Mapping Hypothesis, which proposes that a larger AoA effect appears in deep orthography languages than in shallow orthography languages (Ellis and Lambon Ralph, 2000). Finally, by examining the cross-language AoA effect, researchers can study whether the L2 AoA effect is independent of the L1 AoA effect.

The present database can be developed further. More words can be incorporated, as the currently limited number (no more than 2000 words) imposes constraints on the stimuli selection for researchers. The present database applies only to the L2 studies that use the medium proficiency level of Chinese-English bilingual speakers. It should be also noted that the method of collecting data from a distance has certain limitations, which may affect the data. In future studies, online data collection can be used to further verify the results of the present study.

In summary, the present study provides a large set of norms for variables that are relevant to L2 and L1 AoA research. We hope the present data will be a resource in facilitating experimental research in this field.

### Characteristics of the Word Database

The database is available as a source of Supplementary Material. The first Excel file contains English words that are included on two sheets: in the first sheet (1835 English Words), the L2 AoA ratings are stable and available, while for the words in the second sheet (81 English Words), the L2 AoA ratings are not available for use. The following 17 columns are in the first file.

(1) The word number.
(2) The English word.
(3) The age of acquisition of the second language (L2AoA).
(4) The percentage of participants who know the English word (percent_L2AoA_ recognition).
(5) The number of participants who know the English word (number_L2AoA_ recognition).
(6) The time at which the learner was exposed to a given English word (L2 AoL).
(7) The familiarity data of the second language (L2Fam).
(8) The percentage of participants who provided the familiarity value of the English word (percent_ L2Fam).
(9) The number of participants who provided the familiarity value of the English word (number_ L2Fam).
(10) The concreteness data of the second language (L2Con, from Brysbaert et al., 2014).
(11) The word frequency counts per million from the SUBTLEX-UK corpus (L2Fre/million, van Heuven et al., 2014).
(12) The length of the second language word (L2Len).
(13) The dominant Chinese translation for the English word (dom_trans).
(14) The number of dominant Chinese translations provided by the participants (num_dom_trans).
(15) The number of all Chinese translations provided by the participants (num_all_trans).
(16) The percentage of the dominant translations (percent_dom_trans).
(17) Other translations provided by participants (other_trans).

The second Excel file provides the information on Chinese two-character words and contains the following 15 columns.

(1) The word number.
(2) The Chinese two-character word.
(3) The age of acquisition of the first language (L1AoA).
(4) The percentage of participants who know the Chinese word (percent_L1AoA_ recognition).
(5) The number of participants who know the Chinese word (number_L1AoA_ recognition).
(6) The data source of the L1 AoA data (L1AoA Source).
(7) The familiarity data of the first language (L1Fam).
(8) The percentage of participants who provided the familiarity value of the Chinese word (percent_ L1Fam).
(9) The number of participants who provided the familiarity value of the Chinese word (number_ L1Fam).
(10) The data source of L1Fam data (L1Fam Source).
(11) The concreteness data of the Chinese word (L1Con).
(12) The percentage of participants who provided the concreteness value of Chinese word (percent_ L1Con)
(13) The number of participants who provided the concreteness value of Chinese word (number_ L1Con).
(14) The number of strokes of the Chinese word (L1 Strokes).
(15) The word frequency counts per million from the SUBTLEX-CH-WF corpus (L1Fre/million, Cai and Brysbaert, 2010).

## Data Availability Statement

The raw data supporting the conclusions of this article will be made available by the authors, without undue reservation.

## Ethics Statement

The studies involving human participants were reviewed and approved by the Ethics Committee of the Faculty of Psychology, Beijing Normal University. The patients/participants provided their written informed consent to participate in this study.

## Author Contributions

JW collected and performed the data analysis. Both authors wrote and edited the manuscript, contributed to the article, and approved the submitted version.

## Conflict of Interest

The authors declare that the research was conducted in the absence of any commercial or financial relationships that could be construed as a potential conflict of interest.
